# How do EQ-5D-3L and EQ-5D-5L compare in a Swedish total hip replacement population?

**DOI:** 10.1080/17453674.2020.1746124

**Published:** 2020-04-02

**Authors:** Ted Eneqvist, Szilárd Nemes, Johan Kärrholm, Kristina Burström, Ola Rolfson

**Affiliations:** a Swedish Hip Arthroplasty Register, Gothenburg;; b Department of Orthopedics, Institute of Clinical Sciences, the Sahlgrenska Academy, University of Gothenburg, Gothenburg;;; c Department of Orthopedics, Sahlgrenska University Hospital Gothenburg;; d Health Outcomes and Economic Evaluation Research Group, Stockholm Centre for Healthcare Ethics, Department of Learning, Informatics, Management and Ethics, Karolinska Institutet, Stockholm;; e Equity and Health Policy Research Group, Department of Public Health Sciences, Karolinska Institutet, Stockholm;; f Health Care Services, Region Stockholm, Stockholm, Sweden

## Abstract

Background and purpose — To better detect small changes in postoperative outcome following total hip replacement (THR), the Swedish Hip Arthroplasty Register (SHAR) has decided to change from the EQ-5D-3L (3L) to the EQ-5D-5L (5L). To enable comparison of results obtained with use of the 2 versions of EQ-5D, transferal of results between the questionnaires used is necessary. We assessed the measurement properties of the EQ-5D-5L compared with the EQ-5D-3L, preoperatively and 1-year postoperatively in a Swedish THR population.

Patients and methods — Patients eligible for elective THR during 2015 in Western Sweden were invited to the study. With a 2-week separation, the 3L and 5L questionnaires were administered to patients before and 1 year after surgery. Comparing the 2 versions of the EQ-5D, we investigated redistribution of responses, ceiling and floor effects, EQ VAS correlations (Spearman’s rank correlation coefficient, r_s_), and EQ VAS scores for different severity levels by dimension (univariable ordinary least square regression).

Results — The additional severity levels of the 5L version were frequently used on both measurement occasions (preoperative mobility 5%, self-care 17%, usual activities 20%, pain 5% and anxiety 3%, postoperative mobility 6%, self-care 5%, usual activities 8%, pain 9%, and anxiety 5%). Ceiling effects of the 3L version diminished overall by 7% using the 5L version. The correlations between the 2 EQ VAS scores obtained with the 3L and 5L instruments were strong both pre- (r_s_ = 0.71) and postoperatively (r_s_ = 0.87). Estimated EQ VAS scores for different levels of severity were consistent for all dimensions except for the mobility dimension of the preoperative 5L version and the anxiety dimension in the postoperative 5L version.

Interpretation — Our findings support that the 5L has a higher resolution than the 3L version regarding description of health-related quality of life in patients undergoing THR in Sweden. The EQ VAS scores for different levels of severity agree well between the EQ-5D versions. This could potentially be used to develop a crosswalk value set for transforming 3L to 5L responses in this patient group.

1 of the most commonly used HRQoL instruments is the EQ-5D-3L (3L), which has 5 dimensions (*mobility, self-care, usual activities, pain/discomfort, and anxiety/depression*) and 3 severity levels (*no problems, some problems,* and *confined to bed* [mobility], *unable* [self-care, usual activities] and *severe problems* [pain/discomfort, anxiety/depression]) (Rabin and Charro 2001). It is a short questionnaire and recognized as valid for use in many disease groups and for many conditions (Buchholz et al. 2018), including total hip replacement (THR) patients (Rabin and Charro 2001, Devlin et al. 2013). However, 3L has been questioned due to profound ceiling effects, low sensitivity, and the lack of descriptive richness. As it has limited ability to measure small but clinically relevant changes in the outcome following interventions, its usefulness to assess interventions has been debated (Sullivan et al. 2005). These limitations have been reported for both the general population and specific patient groups (Sullivan et al. 2005, Janssen et al. 2013, Buchholz et al. 2018), including THR patients (Conner-Spady et al. 2015, Greene et al. 2015). Among THR patients, the 3L particularly exhibits difficulties in assessing outcome in the mobility dimension, since the options *no problems, some problems,* and *confined to bed* limit its use in describing the limitations in mobility commonly experienced by patients with hip disorders. These patients typically experience limping, limited range of hip joint motion, and impaired walking capacity—and often require different aids for mobility—but they are seldom confined to bed. Similarly, the response levels for the dimensions self-care and usual activities (*no problems, some problems,* and *unable*) limit the range of responses for individuals with moderate to severe disability (Wolfe and Hawley 1997, Conner-Spady et al. 2015).

In response to the critique of the 3L, the 5L, which has 5 severity levels (*no problems* [mobility, self-care, usual activities]/*no pain* [pain]/*not anxious or depressed* [anxiety/depression]; *some problems* [mobility, self-care, usual activities]/*slight pain* [pain]/*slightly* [anxiety/depression]; *moderate problems* [mobility, self-care, usual activities]/*moderate pain* [pain]/*moderately* [anxiety/depression]; *severe problems* [mobility, self-care, usual activities]/*severe pain* [pain]/*severely* [anxiety/depression]; *confined to bed* [mobility]/*unable to wash or dress* [self-care]/*unable to do* [usual activities]/*extreme pain* [pain]/*extremely* [anxiety/depression]) has been developed (Herdman et al. 2011). The purpose is that the increased number of response levels provides a more accurate profile of the patient’s health. 5L has been compared with the 3L in several studies and has been reported to be valid, to decrease ceiling effects, and to increase discriminatory power in multiple populations (Pickard et al. 2007, Janssen et al. 2013, Scalone et al. 2013, Hinz et al. 2014, Buchholz et al. 2018), including THR patients (Conner-Spady et al. 2015, Greene et al. 2015).

We asked participants to complete the 3L and 5L with 2 weeks’ separation, both preoperatively and 1 year following THR. Comparing the 2 versions of the EQ-5D, we investigated response rates, redistribution into other severity levels, ceiling and floor effects, EQ VAS score correlations, and EQ VAS scores for different severity levels for all 5 dimensions.

## Patients and methods

### EQ-5D-3L and EQ-5D-5L

In the 3L descriptive system an individual’s health state is composed of 1 level for each dimension, so if an individual answers level 1 on each dimension, the health profile is “11111” (full health). The 3L descriptive system yields 243 possible health states (3^5^). In the 5L, the severity levels are: no, slight, moderate, severe, and extreme problems yielding 3,125 unique health states (5^5^). The EQ-5D index is an overall measure of HRQoL, which is calculated by applying weights given by a specific value set. The derived value sets differ between the 3L and 5L, so the indexes are not directly comparable between the 3L and 5L. The 3L is part of the standard follow-up procedures of patients both pre- and postoperatively in several arthroplasty registries (Devlin et al. 2010, Rolfson et al. 2011, Lawless et al. 2012, Greene et al. 2015, LROI report 2016). In 2017, the Swedish Hip Arthroplasty Register (SHAR) started to collect PROMs using the 5L instrument (Kärrholm et al. 2018). Both EQ-5D questionnaires comprise a visual analog scale (EQ VAS), where the patient assesses his/her overall health status from 0 (worst imaginable health state) to 100 (best imaginable health state).

### Patient selection

**Figure 1.**
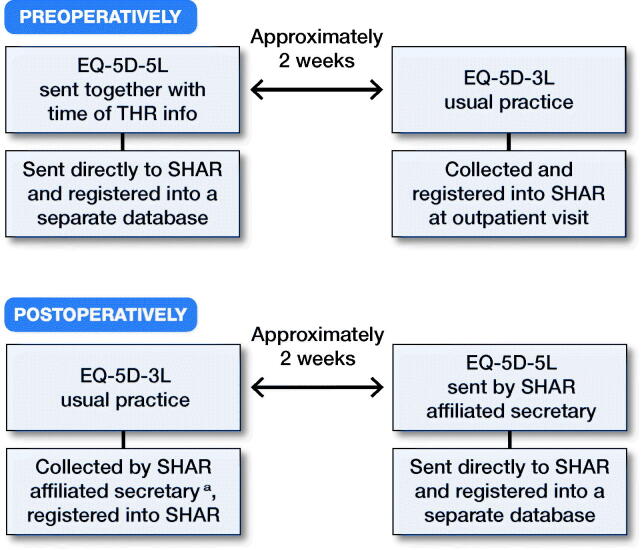
Preoperative and 1-year postoperative procedures for collecting the EQ-5D-3L and -5L questionnaires. **^a^**Non-respondents were reminded after 1 month according to regular procedure.

We invited all patients eligible for THR during 2015 on the basis of primary hip OA at any of the 7 publicly funded hospitals performing THR in the Western region of Sweden. The included hospitals were: Sahlgrenska University Hospital, Södra Älvsborg Hospital, Kungälv Hospital, Norra Älvsborg Hospital, Alingsås Hospital, Skövde Hospital (Kärnsjukhuset), and Lidköping Hospital. For patients on the waiting list for THR, the standard care includes a preparatory preoperative visit to the hospital. At least 2 weeks prior to the preoperative visit, waiting list coordinators at the respective hospitals sent a letter to the patients including general information regarding their upcoming surgery. Patients were informed about the study in this letter and invited to participate. The letter included the 5L version of the EQ-5D questionnaire and a pre-addressed return envelope. Upon completion of the 5L version of the EQ-5D, the questionnaire was sent directly to SHAR and registered in a separate 5L database. No reminders were sent out. Following standard practice of the routine PROMs program of the SHAR, all hospitals ask patients to complete the 3L questionnaire at the preoperative visit (Clement et al. 2011) (Figure 1). 1 year following the index procedure, the SHAR-affiliated secretary at each hospital sent the postoperative 3L questionnaire to all patients. Responses were returned to the hospitals and registered in the SHAR PROMs database by administrative staff. Non-respondents were reminded after 1 month according to regular procedure. Patients received the 5L version together with information about the study and an envelope pre-addressed to SHAR 2 weeks after the 1-year postoperative 3L responses had been registered. When received at SHAR the 5L data was entered to the 5L database by administrative staff (Figure 1).

## Statistics

### Response rates

We calculated response rates for the pre- and postoperative 3L and 5L versions of the questionnaires for the whole group of patients and for each hospital. We compared differences in response rates between the 3L and 5L questionnaires, both pre- and postoperatively.

### Redistribution of responses

**Figure 2.**
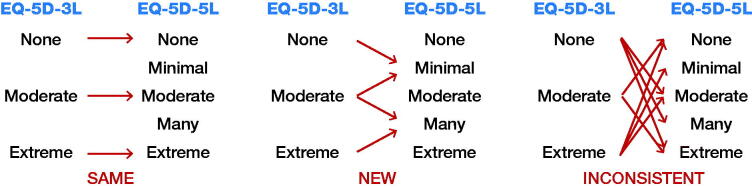
Possible redistribution of responses between EQ-5D-3L and -5L. Inconsistency = choosing an answer 2 levels from the first.

For patients who completed both 3L and 5L questionnaires preoperatively (n = 524) or 1 year postoperatively (n = 508), the responses of the severity level were compared by dimension. The responses were defined as the same, new, or inconsistent. The same answer was defined as the same level between the 2 questionnaires, for example level 1 in 3L and level 1 in 5L. New was defined as 1 level change between the questionnaires, for example answer at level 3 in 3L and level 2 in 5L. Inconsistent was defined as change by 2 severity levels or more between the questionnaires (Janssen et al. 2008), for example answer at level 3 in 3L and at level 1 in 5L (Figure 2).

### Ceiling and floor effects

For the patients who completed both 3L and 5L questionnaires preoperatively or 1 year postoperatively, we calculated the proportion of responses of no problems by separate dimensions and overall to investigate ceiling effects. Similarly, floor effects were investigated by calculating the proportion of patients with extreme problems in separate dimensions and overall. Ceiling and floor effects were considered present if > 15% of the patients reported the best (ceiling) or worst (floor) severity levels (Lim et al. 2015).

### Strength of the association and agreement between EQ-5D-3L and EQ-5D-5L EQ VAS score

To assess and illustrate the agreement of EQ VAS measurements with the 2 EQ VAS scores obtained with the EQ-5D-3L and EQ-5D-5L questionnaires for patients who completed both 3L and 5L questionnaires preoperatively and 1 year postoperatively, we used Bland–Altman plots for differences (Bland and Altman 2010).

To determine the convergence of the EQ VAS scores obtained by the 3L and 5L questionnaires, we used Spearman’s rank correlation coefficient (rs). The strength of the correlations between the 2 questionnaire EQ VAS scores was defined as absent (r_s_ < 0.20), weak (0.20 ≤ r_s_ < 0.35), moderate (0.35 ≤ r_s_ < 0.50), or strong (r_s_ ≥ 0.50) (Myers and Well 2003).

### EQ VAS scores

For the patients who completed both 3L and 5L questionnaires preoperatively or 1 year postoperatively, we used univariable ordinary least squares (OLS) regression models to estimate EQ VAS scores for the different levels of severity of each dimension. The preoperative EQ VAS score was regressed onto the preoperative 3L and 5L dimensions in separate computations. The same calculations with use of postoperative data were repeated for estimation of the postoperative EQ VAS scores.

Each dimension’s level 1 was defined as reference, and the estimated regression coefficients denote the mean difference in EQ VAS scores between patients who reported level 2 and level 1, and level 3 and level 1 and so on. The premise with the regression analysis was that coefficients should have negative signs, and the magnitude of coefficients should increase with the levels.

All statistical analyses were performed in R (R Foundation for Statistical Computing, Vienna, Austria).

## Ethics, funding, and potential conflicts of interests

Ethical review approval was obtained from the Regional Ethical Review Board in Gothenburg, Sweden, registration number 516-14. TE has received funding for the study from the Felix Neubergh foundation. OR and JK were funded by grants from the Swedish state under the agreement between the Swedish government and the county councils, the ALF agreement (ALFGBG-700781). The authors report no conflicts of interest.

## Results

### Response rates

1,567 patients received a THR during 2015 at the included 7 hospitals and were available for the study (Table 1). Of these 1,182 (75%) responded to the preoperative 3L version, 767 (49%) responded to the preoperative 5L version and 524 (33%) answered both questionnaires. For the 1-year follow-up, there were 1,554 patients available as 13 patients were not available due to early reoperation or death. 1,400 (89%) responded to the 1-year 3L version and 508 (32%) responded to the 5L version. Postoperatively, the response rate also differed between the hospitals with an 83–93% response rate for the 3L questionnaire while the proportion of patients who had completed the 5L and both versions ranged from 19% to 49% (Table 2, see Supplementary data).

**Table 1.** Demographics pre- and 1 year postoperatively THR

Factor	n = 1,554
Age, mean (SD)	70 (11)
Female sex, n (%)	878 (57)
ASA, n (%)	
1	340 (22)
2	967 (63)
3	229 (15)
4	1 (0.1)
BMI, mean (SD)	28 (4.8)
EQ VAS preoperatively, mean (SD)	57 (22)
EQ VAS postoperatively, mean (SD)	74 (21)
EQ-5D index preoperatively, mean (SD)	0.40 (0.3)
EQ-5D index postoperatively, mean (SD)	0.76 (0.3)

**Table 3.** Percentage of patients reporting severe problems (floor effect) and no problems (ceiling effect)

	Preoperative patients (n = 524)				Postoperative patients (n = 508)			
	Severe problems (floor effect)		No problems (ceiling effects)		Severe problems (floor effect)		No problems (ceiling effects)	
EQ-5D dimensions	3L	5L	3L	5L	3L	5L	3L	5L
Mobility	0	2	6	2	0	0.2	51	44
Self-care	0.6	0.6	67	30	0.2	0.2	91	76
Usual activities	11	9	27	4	2	1	71	46
Pain/discomfort	44	4	0.6	0.6	5	0.2	39	35
Anxiety/depression	3	0.4	53	39	2	0.2	73	69
Overall	0	0	1	0.4	0	0	32	25

### Redistribution of responses

**Figure 3.**
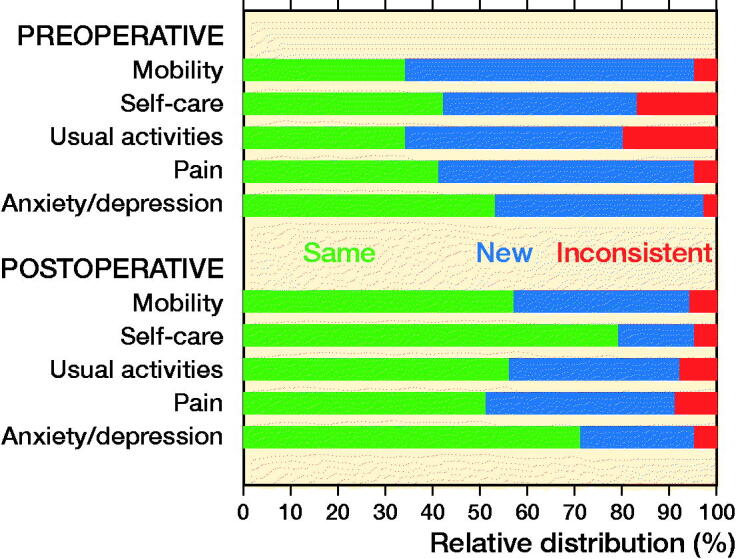
Redistributions of answers between EQ-5D-3L and -5L for all patients.

Preoperatively, a large proportion used new severity levels in the mobility (61%), self-care (41%), usual activities (46%), and pain/discomfort (54%) dimensions in the 5L version (Figure 3). At the 1-year follow-up, patients most frequently reported no problems in all dimensions for both versions (Table 3). Inconsistencies (a response 2 or more levels away from their first response) were reported both pre- and postoperatively. Preoperatively, inconsistencies were most frequently reported in the dimensions self-care (17%) and usual activities (20%). Inconsistencies were less frequent postoperatively compared to preoperatively (Figure 3).

### Ceiling and floor effects

Preoperatively, both versions presented ceiling effects in the self-care and anxiety/depression dimensions, as did usual activities in the 3L version. There were almost no ceiling effects preoperatively (1% for the 3L and 0.4% for the 5L questionnaire). Postoperatively, all dimensions in both 3L and 5L presented ceiling effects but to a lesser extent in the 5L version and the overall ceiling effect differed by 7 percentage units. There were no floor effects except for the 3L pain/discomfort dimension (Table 3).

### Strength of the association and agreement between EQ-5D-3L and EQ-5D-5L EQ VAS score

**Figure 4.**
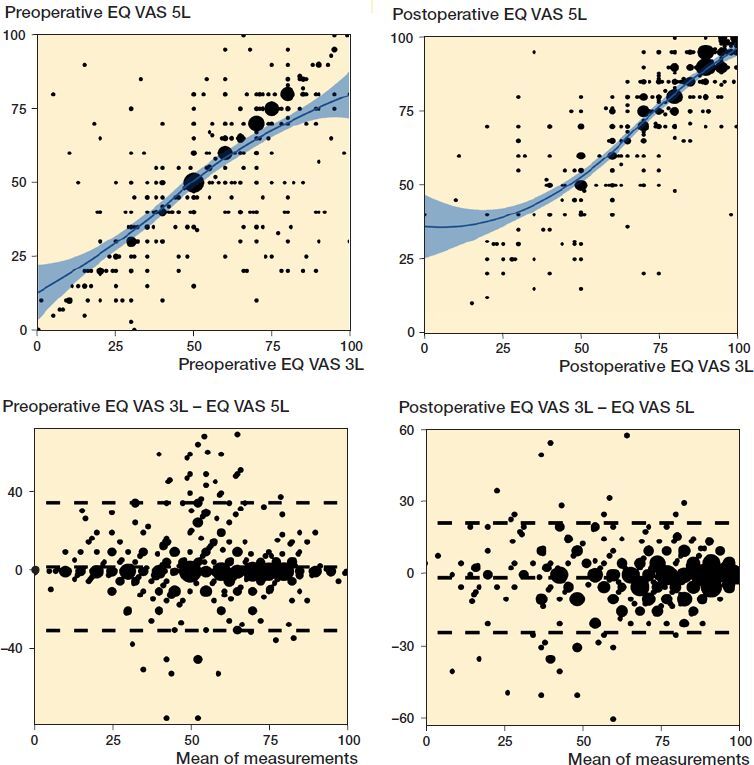
Strength of association and agreement between the EQ VAS scores obtained with the EQ-5D-3L and -5L questionnaires approximately 2 weeks apart. The diameter of the points is proportional to the number of patients reporting that particular score. Note the different scaling of the y-axis in the Bland–Altman plots to improve visibility.

The Bland–Altman plots indicated that there was good agreement between the EQ VAS score measured with the 3L and the 5L questionnaires (Figure 4). Most data points were in between the limits of agreement. This was more accentuated for the postoperative measurement. Highest disagreement was observed at the middle of the EQ VAS scale (at 50), indicating “undecided” patients.

The correlations between the EQ VAS scores obtained with the 3L and 5L from patients who filled out both questionnaires were strong: r_s_ = 0.71 preoperatively and r_s_ = 0.87 postoperatively (Figure 4). Data that disagreed between the 2 measurements tended to have small estimates, which reinforces the indicative value of the analysis.

### EQ VAS scores

The estimated EQ VAS scores and confidence intervals are presented graphically in Figure 5 (see Supplementary data). Due to the rarity of level 4 and 5 responses to the 5L questionnaire, these 2 levels were merged.

With 1 notable exception (mobility), the EQ VAS scores for different levels of severity in the preoperative data conformed well to the expected pattern (Figures 4 and 5, see Supplementary data). Level 1 response on the 5L questionnaire generally resulted in higher estimated EQ VAS than level 1 response on the 3L questionnaire. Level 2 responses on the 5L questionnaire were positioned between level 1 and level 2 on the 3L version. Level 3 on the 5L and level 2 on the 3L version generally resulted in similar scores. The merged level 4 and level 5 responses on the 5L version mostly took values close to the level 3 response of the 3L version.

## Discussion

The 5L was introduced to improve the instrument’s sensitivity and to reduce ceiling effects, as compared with the 3L (Herdman et al. 2011). Our study confirms this intention in patients with hip joint disorders before and 1 year after THR. A vast majority of patients used a new response option of the 5L version in 1 or more dimensions. Although all dimensions presented some ceiling effects 1 year after surgery, the proportion of patients with “11111” decreased from 32% to 25% with the 5L version. These observations are consistent with previous research investigating measurement properties of 3L and 5L in orthopedic patients (Conner-Spady et al. 2015, Greene et al. 2015). The novelty of our work is the projection of response levels for each dimension on the EQ VAS scores for both versions of the EQ-5D questionnaires. The evolution of increasing or decreasing problems in all EQ-5D dimensions was to a great extent mirrored in the self-rated EQ VAS. With 1 exception, the scores for different levels of severity had intuitive order within and between the 2 different versions. The assumption that scores follow a consistent pattern, i.e., that scores for level 1 of the 5L would be higher than the corresponding level for the 3L, is consistent with developing studies of 5L value sets (Devlin et al. 2018). Scores for level 3 on the 5L were generally similar to corresponding mid-level of the 3L version. The 5L level 4/5 scores mostly had similar values as level 3 in the 3L version. We expected this pattern since the 2 levels had to be collapsed as the extreme severity levels were rarely used in our data.

Value set studies have demonstrated that level 5 in the 5L version commonly have higher weights than level 3 in the 3L version (Leidl and Reitmeir 2017, Devlin et al. 2018). However, it would not be appropriate to compare the scores provided in our study with valuation studies as methodologies differ vastly (Oppe et al. 2014). Similar to the developers of the German experience-based value set for the 5L, we used EQ VAS to establish scores for the health states (Leidl and Reitmeir 2017). In experience-based valuations, patients value their own current health state as opposed to health states described to them (Burstrom et al. 2014). Due to the limited number of participants, we used univariate regression models for each dimension to estimate how different levels of severity project on the EQ VAS. Nevertheless, it is a strength to have 3L and 5L responses from the same patients, which allows for a unique comparison of severity measured by the EQ VAS of different health states on the same scale.

Similar to our study, Greene et al. (2015) investigated differences in responses between 3L and 5L questionnaires before and 1 to 6 years after THR. The authors stated that the 5L version is “extremely valuable in identifying preoperative health states, but appears a little less so postoperative.” Although not as strong as our results, they reported strong correlations between the EQ VAS reported in the 2 versions of EQ-5D both pre- and postoperatively. Thus, the strong correlation between the 2 versions indicates convergent validity of the 2 EQ VAS versions. This warrants the use of EQ VAS of the 2 versions to compare the influence of different health states on self-assessed health status. Furthermore, convergent validity between the 3L and 5L versions has been well established regarding the health profile (Golicki et al. 2015). We found similar redistribution of responses as reported by Greene et al. (2015) in similar patients. The pre- and postoperative ceiling effects followed a similar pattern to the results presented here, although our results suggest an even more pronounced reduction of the ceiling effect with the 5L.

Conner-Spady et al. (2015) investigated the validity and reliability of the 5L version compared with the 3L version in 176 patients with OA referred for hip or knee replacement with a similar study set-up to ours. Compared with our preoperative result, these authors found a smaller proportion of patients who used new severity levels in the 5L version for all 5 dimensions. The authors concluded that the added levels in the 5L version provided stronger evidence of validity compared with the 3L version for patients with hip and knee OA referred for total joint replacement, in particular for the 3 dimensions that are particularly relevant to this patient population: mobility, usual activities, and pain/discomfort.

The studies by Green et al. (2015) and Connor-Spady et al. (2015) suggest that the 5L has the ability to better discriminate health states in patients eligible for THR. This feature may improve health outcome assessment following THR. THR is predominantly an elective procedure with the main purpose to reduce pain and gain mobility and HRQoL. If the dimensions of pain/discomfort, usual activities, and mobility are not improved by the intervention, it is likely that the patient is not satisfied with the outcome of the procedure (Anakwe et al. 2011, Clement et al. 2011). Diminished ceiling effects enable a more accurate outcome assessment so that changes in care can be better monitored.

The most commonly used method to investigate differences between the 3L and 5L versions is to administer both questionnaires in the same session with demographic and/or other questionnaires in between the 2 versions (Janssen et al. 2013, Scalone et al. 2013, Craig et al. 2014). However, Janssen et al. (2008) found that patients tended to avoid using the intermediate level in the 5L questionnaire if the 3L version was administered first at the same sitting. To possibly avoid this bias, we decided to administer the 2 questionnaires with a 2-week gap in between. Similar to Greene et al. (2015), we found higher rates of inconsistent responses in the preoperative pain/discomfort and mobility dimensions than seen in other studies (Janssen et al. 2008, Scalone et al. 2013). Due to natural fluctuations in hip symptoms, the 2-week time span likely contributed to the inconsistent responses both in separate dimensions and in EQ VAS. This is a limitation that could be caused by properties of the instrument itself and/or by a true change in health state.

We are aware that HRQoL measures such as EQ VAS are subjective measures and show temporal variability. Thus, we cannot expect that EQ VAS measurement at different time points should have complete agreement. However, estimates in our study are based on the presumption that most of variability in EQ VAS score at follow-up can be explained by the preceding measurement. As the 2 measurements were only 2 weeks apart there should not be substantial disagreement/or trends in disagreement in the Bland–Altman plots, and the strength of relationship between the EQ VAS scores at the 2 time points should be strong.

There is also a possible bias in the preoperative selection of patients, since no reminder was sent out after the preoperative collection of the 5L version. This was not considered possible because of too short a time period until surgery. A reminder would therefore be likely to reach the patient after the THR procedure, which most certainly would have influenced the health status and the answers from the patient. Nonetheless, the health profile might have differed between the responders and non-responders. If so, we think that this difference is small when measured with EQ VAS, with comparatively low impact on the results of this study.

Another limitation is that we did not randomize the order in which the respondents completed the 2 versions. There was a larger redistribution of responses preoperatively when the patients responded to the 5L version first compared with postoperatively when the order of questionnaires was the opposite. However, we believe this was not a result of the order of responses but rather an effect of the improved health state with a large proportion having no problems.

The response rate for the 5L version was generally much lower than for the 3L version. We do not interpret this as a reluctance among patients to respond to a more comprehensive questionnaire. The 3L version was collected through a well-established routine as opposed to additional collection of 5L. We have no information on whether patients actually received the 5L version. Logistical challenges at the participating hospitals for distributing the additional 5L version may well explain the poorer response rate as exemplified by large differences between the different hospitals. Both the pre- and postoperative response rates of the 3L version were in all hospitals, except 1, close to the average response rate of the 3L in the SHAR (Kärrholm et al. 2018). This is a considerably higher response rate to the questionnaire than in similar studies (Conner-Spady et al. 2015, Greene et al. 2015).

## Conclusion

The results indicate that 5L describes HRQoL in more detail in patients undergoing THR in Sweden. The EQ VAS scores for different levels of severity agree well between the 3L and the 5L questionnaires. Our research will be directed into the development of a crosswalk value set for transforming 3L to 5L responses in this patient group to be able to follow up over time.

## Supplementary Material

Supplemental Material

## Supplementary data

Table 2 and Figure 5 are available as supplementary data in the online version of this article, http://dx.doi.org/10.1080/17453674.2020.1746124
