# Supplementary material for: How do EQ-5D-3L and EQ-5D-5L compare in a Swedish total hip replacement population?
Source: Acta Orthop. 2020 Apr 2;91(3):272–8. doi: 10.1080/17453674.2020.1746124 (PMC8023878; doi:10.1080/17453674.2020.1746124)
Supplement: Supplemental Material [file IORT_A_1746124_SM4427.pdf]

Supplementary data

Table 2. Number of elective total hip replacements at the participating hospitals in 2015 and response rates to the EQ-5D-3L and -5L questionnaires. Values are frequency (%)

	Preoperative				Postopoperative			
Clinic	Total	3L	5L	3L and 5L	Total	3L	5L	3L and 5L
Borås	99	87 (88)	67 (68)	65 (66)	99	90 (91)	49 (49)	49 (49)
SU/Mölndal	419	289 (69)	204 (49)	131 (31)	414	381 (92)	143 (35)	143 (35)
Skövde	127	38 (30)	80 (63)	7 (6)	125	104 (83)	43 (34)	43 (34)
Uddevalla	313	235 (75)	161 (51)	109 (35)	310	275 (89)	101 (33)	101 (33)
Lidköping	261	226 (87)	97 (37)	81 (31)	259	227 (88)	65 (25)	65 (25)
Kungälv	165	157 (95)	98 (59)	93 (56)	165	154 (93)	72 (44)	72 (44)
Alingsås	183	150 (82)	60 (33)	38 (21)	182	169 (93)	35 (19)	35 (19)
All hospitals	1,567	1,182 (75)	767 (49)	524 (33)	1,554	1,400 (90)	508 (33)	508 (33)

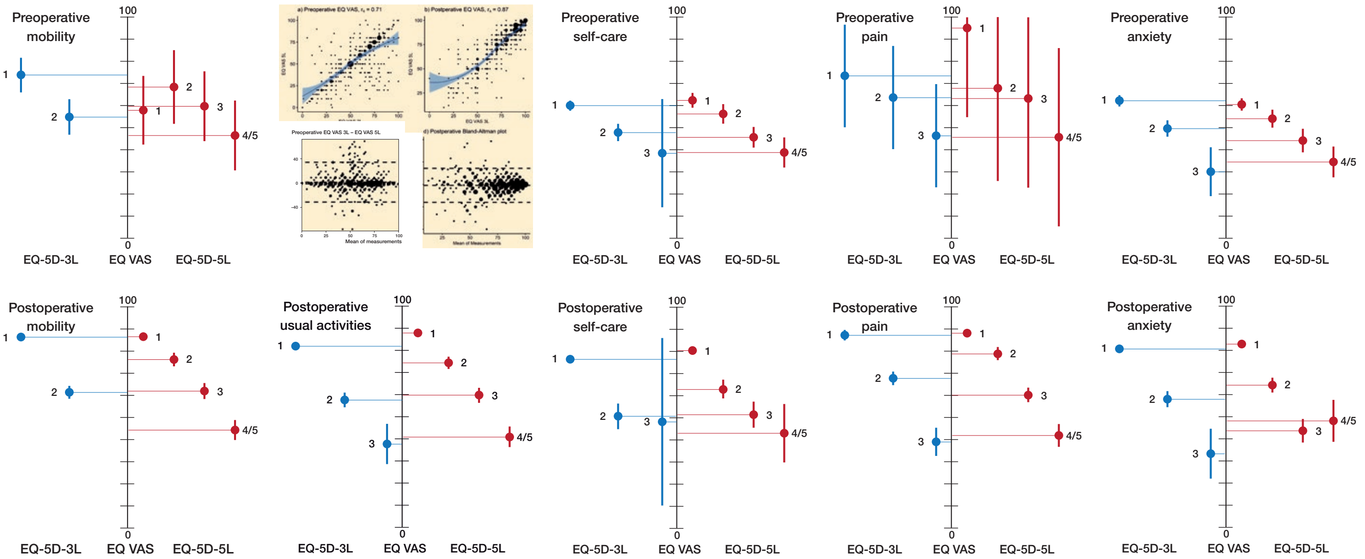

Figure 5. Pre- and postoperative regression of the EQ-5D-3L and -5L questionnaires with EQ VAS by dimensions. All calculations made from questionnaires from patients who filled out both questionnaires
